# Poly[tetra­kis(μ-cyclo­hexa­ne-1,4-di­carboxyl­ato)di-μ-hydroxido-penta­zinc(II)]

**DOI:** 10.1107/S1600536809051666

**Published:** 2009-12-04

**Authors:** Jin-Xi Chen, Wei-Wei Meng

**Affiliations:** a School of Chemistry and Chemical Engineering, Southeast University, Nanjing 211189, People’s Republic of China

## Abstract

In the title coordination polymer, [Zn_5_(μ_3_-OH)_2_(1,4-CDC)_4_]_*n*_ (1,4-CDCH_2_ = 1,4-cyclo­hexa­nedicarboxylic acid) or [Zn_5_(C_8_H_10_O_4_)_4_(OH)_2_]_*n*_, the asymmetric unit comprises one half of an octa­hedrally coordinated ZnO_6_ complex unit (site symmetry 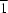) and two five-coordinate ZnO_5_ complex units, together with two μ_3_-bridging hydroxido ligands and four 1,4-CDC ligands (comprising two whole mol­ecules and four inversion-related half-molecules). The ZnO_6_ unit consists of four carboxyl­ate O donors (two bridging) and two hydroxido O donors (both bridging three Zn centres) [Zn—O range 2.065 (3)–2.125 (3) Å]. Each of the ZnO_5_ units [one capped tetra­hedral, the other square-pyrimidal; Zn—O range 1.928 (3)–2.338 (3) Å] has one hydroxido O donor and four carboxyl O donors from three different 1,4-CDC carboxyl­ate O donors (one bridging). Infinite (ZnO)_*n*_ inorganic chains run parallel to the *a* axis and are interconnected by the organic ligands into a three-dimensional structure.

## Related literature

For the structures of related complexes of 1,4-cyclo­hexa­nedicarboxylic acid, see: Liu, Huang *et al.* (2009 ▶); Liu, Zhu *et al.* (2009 ▶); Yang *et al.* (2007 ▶); Du *et al.* (2005 ▶).
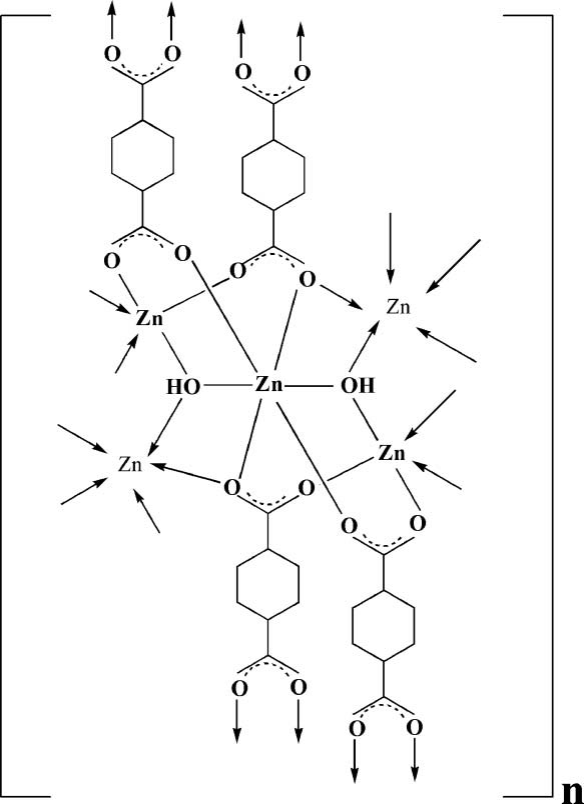


## Experimental

### 

#### Crystal data

[Zn_5_(C_8_H_10_O_4_)_4_(OH)_2_]
*M* *_r_* = 1039.59
Triclinic, 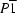

*a* = 8.646 (3) Å
*b* = 10.665 (3) Å
*c* = 11.804 (3) Å
α = 113.915 (3)°
β = 96.307 (3)°
γ = 106.285 (3)°
*V* = 923.6 (5) Å^3^
*Z* = 1
Mo *K*α radiation
μ = 3.28 mm^−1^
*T* = 295 K
0.20 × 0.20 × 0.20 mm

#### Data collection

Rigaku SCXmini diffractometer
3929 measured reflections
3202 independent reflections
2789 reflections with *I* > 2σ(*I*)
*R*_int_ = 0.019

#### Refinement

*R*[*F* ^2^ > 2σ(*F* ^2^)] = 0.038
*wR*(*F* ^2^) = 0.095
*S* = 1.04
3202 reflections
250 parameters
H-atom parameters constrained
Δρ_max_ = 1.11 e Å^−3^
Δρ_min_ = −0.50 e Å^−3^

### 

Data collection: *CrystalClear* (Rigaku, 2005 ▶); cell refinement: *CrystalClear*; data reduction: *CrystalClear*; program(s) used to solve structure: *SHELXS97* (Sheldrick, 2008 ▶); program(s) used to refine structure: *SHELXL97* (Sheldrick, 2008 ▶); molecular graphics: *SHELXTL* (Sheldrick, 2008 ▶); software used to prepare material for publication: *SHELXTL*.

## Supplementary Material

Crystal structure: contains datablocks I, New_Global_Publ_Block. DOI: 10.1107/S1600536809051666/zs2020sup1.cif

Structure factors: contains datablocks I. DOI: 10.1107/S1600536809051666/zs2020Isup2.hkl

Additional supplementary materials:  crystallographic information; 3D view; checkCIF report

## Figures and Tables

**Fig. 1.**
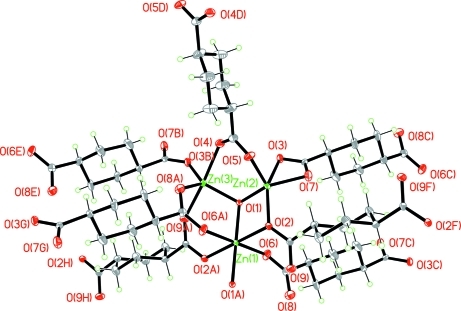
The asymmetric unit of the title compound (I), with displacement ellipsoids drawn at the 30% probability level. For symmetry codes: (A),-x+1,-y+1,-z+1; (B),-x,-y+1,-z+1; (C),-x,-y,-z; (D),-x,-y+2,-z+1; (E), -x,-y-1,-z+1;(F),-x+1,-y+1,-z; (G), x+1, y+1, z+1; (H), x, y,z+1.

**Fig. 2.**
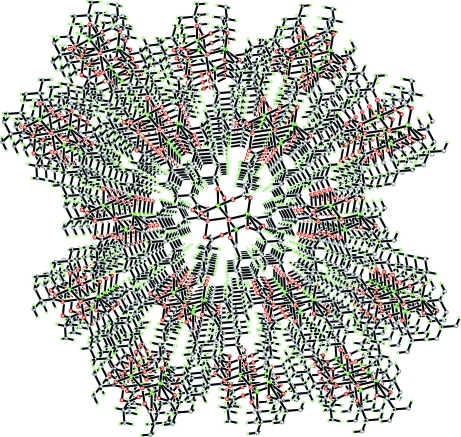
The two-dimensional framework polymer structure of (I).

## supplementary crystallographic information

### Comment

In recent years, new coordination compounds formed from reaction of metals with cyclohexane-1,4-dicarboxylic acid [1,4-CDCH_2_] have attracted much attention (Liu, Huang *et al.*, 2009; Liu, Zhu *et al.*, 2009; Yang *et al.*, 2007; Du *et al.*, 2005). The structure of the title complex from the reaction of this acid with Zn^II^ ion, [Zn_5_(µ_3_-OH)_2_(1,4-CDC)_4_]_n_ (I) has been determined and the structure is reported here.

Compound (I) is a coordination polymer in which the repeating unit lies on a crystallographic inversion centre,the asymmetric unit comprising a half of an octahedraly coordinated Zn atom (Zn1) which lies on the centre, two five-coordinate Zn atoms in (Zn2 and Zn3), general sites, one µ_3_-hydroxido ligand (O1) and two cyclohexane-1,4-dicarboxylate ligands (Fig. 1). One of these 1,4-CDC ligands is complete (associated with donor atoms O6, O8, O3, O7) while the other 1,4-CDC ligand consists of two inversion-related halves (associated with O4, O5 and O2, O9). The ZnO_6_ coordination sphere about Zn1 consists of four carboxylate O donors (two bridging) and two hydroxido O donors (both bridging three Zn centres), [Zn—O bond length range, 2.065 (3)–2.125 (3) Å]. Both Zn2 and Zn3 are five coordinate, Zn2 having a capped tetrahedral stereochemistry, comprising one bridging hydroxyl O donor and four O donors from three different 1,4-CDC ligands (one bridging) [Zn–O bond length and O–Zn–O bond angle ranges, 1.928 (3)–2.338 (3) Å and 58.85 (11)–145.39 (12)° respectively]. The stereochemistry about Zn3 is tetragonal pyramidal with the four basal coordination sites occupied by O donor atoms from three different 1,4-CDC ligands (one bridging) [Zn—O range, 1.938 (3)–2.207 (3) Å], with the axial site occupied by the bridging hydroxido O donor [Zn–O1, 1.977 (3) Å]. The bond angle range is 86.00 (12)–125.97 (12)°. The repeat units form infinite (ZnO)_n_ inorganic chains parallel to the *a*-axis which are interconnected by the organic ligands into a three-dimensional structure (Fig. 2).

### Experimental

An aqueous mixture of cyclohexane-1,4-dicarboxylic acid (0.086 g, 0.5 mmol) and NaOH (0.040 g, 1 mmol) in 8 ml of water was stirred for half an hour. The pH was adjusted to *ca*. 7 with 1M HNO_3_ and (0.147 g, 0.5 mmol) of Zn(NO_3_)_2_ . 6H_2_O  was added and the solution was stirred for half an hour. After adding 3 ml of cyclohexanol, the mixture was transferred into a 23 ml Teflon-lined autoclave and heated at 180° for 120 h. After cooling to room temperature, colorless single crystal blocks were obtained, which were washed with water.

### Refinement

All H atoms were fixed geometrically and treated as riding, with C—H = 0.97 –0.98 Å and with *U*iso(H) = 1.2*U*eq(C).

### Crystal data

**Table:**

[Zn_5_(OH)_2_(C_8_H_10_O_4_)_4_]	*Z* = 1
*M**_r_* = 1039.59	*F*(000) = 526
Triclinic, *P*1	*D*_x_ = 1.869 Mg m^−^^3^
Hall symbol: -P 1	Mo *K*α radiation, λ = 0.71073 Å
*a* = 8.646 (3) Å	Cell parameters from 30 reflections
*b* = 10.665 (3) Å	θ = 3–25°
*c* = 11.804 (3) Å	µ = 3.28 mm^−^^1^
α = 113.915 (3)°	*T* = 295 K
β = 96.307 (3)°	Block, colorless
γ = 106.285 (3)°	0.20 × 0.20 × 0.20 mm
*V* = 923.6 (5) Å^3^	

### Data collection

**Table:**

Rigaku SCXmini diffractometer	*R*_int_ = 0.019
Radiation source: fine-focus sealed tube	θ_max_ = 25.0°, θ_min_ = 2.0°
graphite	*h* = −10→10
ω scans	*k* = −12→9
3929 measured reflections	*l* = −14→13
3202 independent reflections	3 standard reflections every 150 reflections
2789 reflections with *I* > 2σ(*I*)	intensity decay: none

### Refinement

**Table:**

Refinement on *F*^2^	Primary atom site location: structure-invariant direct methods
Least-squares matrix: full	Secondary atom site location: difference Fourier map
*R*[*F*^2^ > 2σ(*F*^2^)] = 0.038	Hydrogen site location: inferred from neighbouring sites
*wR*(*F*^2^) = 0.095	H-atom parameters constrained
*S* = 1.04	*w* = 1/[σ^2^(*F*_o_^2^) + (0.0488*P*)^2^ + 2.2254*P*] where *P* = (*F*_o_^2^ + 2*F*_c_^2^)/3
3202 reflections	(Δ/σ)_max_ < 0.001
250 parameters	Δρ_max_ = 1.11 e Å^−^^3^
0 restraints	Δρ_min_ = −0.50 e Å^−^^3^

### Special details

**Table:**

Geometry. All e.s.d.'s (except the e.s.d. in the dihedral angle between two l.s. planes) are estimated using the full covariance matrix. The cell e.s.d.'s are taken into account individually in the estimation of e.s.d.'s in distances, angles and torsion angles; correlations between e.s.d.'s in cell parameters are only used when they are defined by crystal symmetry. An approximate (isotropic) treatment of cell e.s.d.'s is used for estimating e.s.d.'s involving l.s. planes.
Refinement. Refinement of *F*^2^ against ALL reflections. The weighted *R*-factor *wR* and goodness of fit *S* are based on *F*^2^, conventional *R*-factors *R* are based on *F*, with *F* set to zero for negative *F*^2^. The threshold expression of *F*^2^ > σ(*F*^2^) is used only for calculating *R*-factors(gt) *etc*. and is not relevant to the choice of reflections for refinement. *R*-factors based on *F*^2^ are statistically about twice as large as those based on *F*, and *R*- factors based on ALL data will be even larger.

### Fractional atomic coordinates and isotropic or equivalent isotropic displacement parameters (Å^2^)

**Table:**

	*x*	*y*	*z*	*U*_iso_*/*U*_eq_	
Zn1	0.5000	0.5000	0.5000	0.01886 (17)	
Zn2	0.23318 (6)	0.53614 (5)	0.33384 (4)	0.02004 (14)	
Zn3	0.22931 (6)	0.63639 (5)	0.63471 (4)	0.02095 (15)	
O1	0.2545 (3)	0.4858 (3)	0.4786 (2)	0.0165 (6)	
O2	0.4649 (3)	0.5325 (3)	0.3351 (3)	0.0227 (6)	
O3	−0.0494 (4)	0.3940 (3)	0.2794 (3)	0.0260 (7)	
O4	0.0760 (4)	0.7048 (3)	0.5332 (3)	0.0292 (7)	
O5	0.2146 (4)	0.7268 (3)	0.3889 (3)	0.0329 (8)	
O6	0.4102 (4)	0.2695 (3)	0.3800 (3)	0.0333 (8)	
O7	0.1035 (4)	0.4072 (4)	0.1489 (3)	0.0354 (8)	
O8	0.5888 (4)	0.1755 (3)	0.2861 (3)	0.0367 (8)	
O9	0.6515 (4)	0.4466 (4)	0.2502 (3)	0.0391 (9)	
C1	−0.0356 (5)	0.3514 (5)	0.1658 (4)	0.0216 (9)	
C2	0.4892 (6)	0.5179 (5)	0.1295 (4)	0.0244 (9)	
H2	0.4089	0.5681	0.1453	0.029*	
C3	0.5402 (5)	0.4959 (5)	0.2450 (4)	0.0210 (9)	
C4	−0.1756 (5)	0.2307 (5)	0.0558 (4)	0.0239 (9)	
H4	−0.2808	0.2294	0.0799	0.029*	
C5	0.4451 (6)	0.1700 (5)	0.2982 (4)	0.0266 (10)	
C6	0.3284 (6)	−0.1108 (5)	0.1772 (4)	0.0340 (11)	
H6A	0.3326	−0.1206	0.2556	0.041*	
H6B	0.4337	−0.1087	0.1553	0.041*	
C7	0.4060 (6)	0.3693 (5)	0.0104 (4)	0.0293 (10)	
H7A	0.4817	0.3156	−0.0022	0.035*	
H7B	0.3068	0.3126	0.0236	0.035*	
C8	0.3027 (5)	0.0317 (5)	0.1984 (4)	0.0274 (10)	
H8	0.1995	0.0306	0.2258	0.033*	
C9	0.6414 (6)	0.6136 (5)	0.1087 (4)	0.0330 (11)	
H9A	0.6883	0.7096	0.1827	0.040*	
H9B	0.7258	0.5689	0.1000	0.040*	
C10	−0.1861 (6)	0.2424 (5)	−0.0691 (4)	0.0330 (11)	
H10A	−0.0815	0.2467	−0.0928	0.040*	
H10B	−0.2041	0.3323	−0.0568	0.040*	
C11	0.1180 (6)	0.7593 (5)	0.4592 (4)	0.0281 (10)	
C12	0.0494 (7)	0.8716 (6)	0.4494 (6)	0.0441 (13)	
H12	0.0263	0.8499	0.3590	0.053*	
C13	−0.1518 (7)	0.0883 (5)	0.0363 (5)	0.0425 (13)	
H13A	−0.1540	0.0779	0.1141	0.051*	
H13B	−0.0434	0.0922	0.0200	0.051*	
C14	0.2867 (7)	0.0456 (5)	0.0755 (5)	0.0396 (12)	
H14A	0.3928	0.0582	0.0533	0.047*	
H14B	0.2606	0.1326	0.0894	0.047*	
C15	0.1774 (7)	1.0209 (7)	0.5214 (7)	0.0554 (16)	
H15A	0.2750	1.0237	0.4876	0.066*	
H15B	0.2105	1.0432	0.6107	0.066*	
C16	−0.1133 (7)	0.8624 (6)	0.4875 (7)	0.0518 (15)	
H16A	−0.0976	0.8752	0.5746	0.062*	
H16B	−0.1962	0.7657	0.4319	0.062*	

### Atomic displacement parameters (Å^2^)

**Table:**

	*U*^11^	*U*^22^	*U*^33^	*U*^12^	*U*^13^	*U*^23^
Zn1	0.0198 (3)	0.0240 (4)	0.0140 (3)	0.0118 (3)	0.0024 (3)	0.0077 (3)
Zn2	0.0223 (3)	0.0226 (3)	0.0165 (3)	0.0107 (2)	0.00190 (19)	0.0092 (2)
Zn3	0.0198 (3)	0.0234 (3)	0.0170 (3)	0.0083 (2)	0.00536 (19)	0.0062 (2)
O1	0.0185 (13)	0.0181 (14)	0.0134 (13)	0.0092 (11)	0.0029 (11)	0.0062 (11)
O2	0.0255 (15)	0.0346 (17)	0.0163 (14)	0.0167 (13)	0.0072 (12)	0.0150 (13)
O3	0.0235 (15)	0.0329 (17)	0.0157 (15)	0.0074 (13)	0.0038 (12)	0.0077 (13)
O4	0.0330 (17)	0.0306 (17)	0.0320 (18)	0.0168 (14)	0.0109 (14)	0.0175 (15)
O5	0.0436 (19)	0.0259 (17)	0.045 (2)	0.0222 (15)	0.0220 (16)	0.0213 (15)
O6	0.0372 (19)	0.0199 (16)	0.0304 (18)	0.0063 (14)	0.0105 (15)	0.0020 (14)
O7	0.0249 (17)	0.046 (2)	0.0211 (17)	0.0037 (15)	0.0056 (13)	0.0077 (15)
O8	0.0246 (17)	0.0229 (17)	0.045 (2)	0.0037 (13)	0.0040 (15)	0.0030 (15)
O9	0.051 (2)	0.069 (2)	0.0274 (18)	0.046 (2)	0.0192 (16)	0.0314 (18)
C1	0.019 (2)	0.027 (2)	0.020 (2)	0.0133 (18)	0.0068 (17)	0.0076 (18)
C2	0.036 (2)	0.035 (3)	0.020 (2)	0.024 (2)	0.0138 (19)	0.020 (2)
C3	0.023 (2)	0.029 (2)	0.015 (2)	0.0126 (18)	0.0066 (16)	0.0118 (18)
C4	0.021 (2)	0.026 (2)	0.021 (2)	0.0091 (18)	0.0035 (17)	0.0071 (18)
C5	0.027 (2)	0.025 (2)	0.024 (2)	0.0082 (19)	0.0048 (18)	0.009 (2)
C6	0.047 (3)	0.026 (2)	0.017 (2)	0.005 (2)	−0.004 (2)	0.006 (2)
C7	0.033 (2)	0.032 (3)	0.028 (2)	0.011 (2)	0.0070 (19)	0.018 (2)
C8	0.023 (2)	0.023 (2)	0.025 (2)	0.0057 (18)	0.0033 (18)	0.0029 (19)
C9	0.036 (3)	0.037 (3)	0.024 (2)	0.009 (2)	0.002 (2)	0.017 (2)
C10	0.046 (3)	0.020 (2)	0.020 (2)	0.006 (2)	−0.002 (2)	0.0038 (19)
C11	0.030 (2)	0.024 (2)	0.033 (3)	0.0117 (19)	0.010 (2)	0.014 (2)
C12	0.062 (4)	0.038 (3)	0.059 (4)	0.033 (3)	0.034 (3)	0.032 (3)
C13	0.047 (3)	0.031 (3)	0.038 (3)	0.006 (2)	−0.011 (2)	0.016 (2)
C14	0.043 (3)	0.020 (2)	0.040 (3)	−0.001 (2)	−0.009 (2)	0.012 (2)
C15	0.042 (3)	0.056 (4)	0.084 (5)	0.029 (3)	0.019 (3)	0.038 (4)
C16	0.044 (3)	0.039 (3)	0.080 (4)	0.018 (3)	0.017 (3)	0.032 (3)

### Geometric parameters (Å, °)

**Table:**

Zn1—O1	2.065 (3)	C5—C8	1.528 (6)
Zn1—O1^i^	2.065 (3)	C6—C8	1.521 (6)
Zn1—O2^i^	2.116 (3)	C6—C10^iii^	1.532 (6)
Zn1—O2	2.116 (3)	C6—H6A	0.9700
Zn1—O6	2.125 (3)	C6—H6B	0.9700
Zn1—O6^i^	2.125 (3)	C7—C9^iv^	1.517 (6)
Zn2—O5	1.928 (3)	C7—H7A	0.9700
Zn2—O1	1.993 (3)	C7—H7B	0.9700
Zn2—O2	2.013 (3)	C8—C14	1.513 (7)
Zn2—O7	2.019 (3)	C8—H8	0.9800
Zn2—O3	2.338 (3)	C9—C7^iv^	1.517 (6)
Zn3—O8^i^	1.939 (3)	C9—H9A	0.9700
Zn3—O3^ii^	1.971 (3)	C9—H9B	0.9700
Zn3—O1	1.977 (3)	C10—C6^iii^	1.532 (6)
Zn3—O4	2.151 (3)	C10—H10A	0.9700
Zn3—O9^i^	2.207 (3)	C10—H10B	0.9700
O2—C3	1.290 (5)	C11—C12	1.516 (6)
O3—C1	1.261 (5)	C12—C15	1.480 (8)
O4—C11	1.263 (5)	C12—C16	1.513 (8)
O5—C11	1.260 (5)	C12—H12	0.9800
O6—C5	1.250 (5)	C13—C14^iii^	1.530 (6)
O7—C1	1.261 (5)	C13—H13A	0.9700
O8—C5	1.254 (5)	C13—H13B	0.9700
O9—C3	1.226 (5)	C14—C13^iii^	1.530 (6)
C1—C4	1.495 (6)	C14—H14A	0.9700
C2—C3	1.514 (5)	C14—H14B	0.9700
C2—C7	1.529 (6)	C15—C16^v^	1.532 (7)
C2—C9	1.535 (6)	C15—H15A	0.9700
C2—H2	0.9800	C15—H15B	0.9700
C4—C13	1.518 (6)	C16—C15^v^	1.532 (7)
C4—C10	1.524 (6)	C16—H16A	0.9700
C4—H4	0.9800	C16—H16B	0.9700
			
O1—Zn1—O2	80.38 (10)	O8—C5—C8	115.7 (4)
O1—Zn1—O6	87.49 (11)	C8—C6—C10^iii^	110.4 (4)
O2—Zn1—O6	89.32 (12)	C8—C6—H6A	109.6
O1—Zn1—O6^i^	92.51 (11)	C10^iii^—C6—H6A	109.6
O2—Zn1—O6^i^	90.68 (12)	C8—C6—H6B	109.6
O6—Zn1—O6^i^	180.00 (12)	C10^iii^—C6—H6B	109.6
O1—Zn1—Zn2^i^	140.05 (7)	H6A—C6—H6B	108.1
O2—Zn1—Zn2^i^	139.33 (8)	C9^iv^—C7—C2	111.9 (4)
O6—Zn1—Zn2^i^	88.74 (9)	C9^iv^—C7—H7A	109.2
O5—Zn2—O1	111.19 (13)	C2—C7—H7A	109.2
O5—Zn2—O2	115.65 (13)	C9^iv^—C7—H7B	109.2
O1—Zn2—O2	84.68 (11)	C2—C7—H7B	109.2
O5—Zn2—O7	108.81 (15)	H7A—C7—H7B	107.9
O1—Zn2—O7	129.58 (13)	C14—C8—C6	110.5 (4)
O2—Zn2—O7	104.36 (12)	C14—C8—C5	106.2 (4)
O5—Zn2—O3	98.81 (13)	C6—C8—C5	114.1 (4)
O1—Zn2—O3	85.57 (11)	C14—C8—H8	108.6
O2—Zn2—O3	145.41 (12)	C6—C8—H8	108.6
O7—Zn2—O3	58.86 (11)	C5—C8—H8	108.6
O8^i^—Zn3—O3^ii^	118.92 (13)	C7^iv^—C9—C2	111.0 (4)
O8^i^—Zn3—O1	114.91 (13)	C7^iv^—C9—H9A	109.4
O3^ii^—Zn3—O1	125.97 (12)	C2—C9—H9A	109.4
O8^i^—Zn3—O4	93.78 (14)	C7^iv^—C9—H9B	109.4
O3^ii^—Zn3—O4	86.00 (12)	C2—C9—H9B	109.4
O1—Zn3—O4	94.98 (11)	H9A—C9—H9B	108.0
O8^i^—Zn3—O9^i^	92.49 (15)	C4—C10—C6^iii^	111.1 (4)
O3^ii^—Zn3—O9^i^	84.37 (13)	C4—C10—H10A	109.4
O1—Zn3—O9^i^	89.19 (12)	C6^iii^—C10—H10A	109.4
O4—Zn3—O9^i^	170.20 (13)	C4—C10—H10B	109.4
Zn3—O1—Zn2	111.16 (13)	C6^iii^—C10—H10B	109.4
Zn3—O1—Zn1	110.07 (12)	H10A—C10—H10B	108.0
Zn2—O1—Zn1	98.36 (11)	O5—C11—O4	124.9 (4)
C3—O2—Zn2	132.3 (3)	O5—C11—C12	115.1 (4)
C3—O2—Zn1	125.5 (3)	O4—C11—C12	120.0 (4)
Zn2—O2—Zn1	96.09 (11)	C15—C12—C16	112.4 (5)
C1—O3—Zn3^ii^	137.0 (3)	C15—C12—C11	110.3 (5)
C1—O3—Zn2	84.3 (2)	C16—C12—C11	113.4 (4)
Zn3^ii^—O3—Zn2	138.63 (14)	C15—C12—H12	106.8
C11—O4—Zn3	125.9 (3)	C16—C12—H12	106.8
C11—O5—Zn2	118.8 (3)	C11—C12—H12	106.8
C5—O6—Zn1	142.7 (3)	C4—C13—C14^iii^	112.1 (4)
C1—O7—Zn2	99.0 (3)	C4—C13—H13A	109.2
C5—O8—Zn3^i^	119.7 (3)	C14^iii^—C13—H13A	109.2
C3—O9—Zn3^i^	140.7 (3)	C4—C13—H13B	109.2
O7—C1—O3	117.8 (4)	C14^iii^—C13—H13B	109.2
O7—C1—C4	121.1 (4)	H13A—C13—H13B	107.9
O3—C1—C4	121.0 (4)	C8—C14—C13^iii^	112.6 (4)
C3—C2—C7	110.1 (3)	C8—C14—H14A	109.1
C3—C2—C9	110.3 (4)	C13^iii^—C14—H14A	109.1
C7—C2—C9	110.4 (4)	C8—C14—H14B	109.1
C3—C2—H2	108.6	C13^iii^—C14—H14B	109.1
C7—C2—H2	108.6	H14A—C14—H14B	107.8
C9—C2—H2	108.6	C12—C15—C16^v^	111.8 (5)
O9—C3—O2	122.9 (4)	C12—C15—H15A	109.2
O9—C3—C2	119.3 (4)	C16^v^—C15—H15A	109.2
O2—C3—C2	117.8 (4)	C12—C15—H15B	109.2
C1—C4—C13	106.4 (4)	C16^v^—C15—H15B	109.2
C1—C4—C10	115.0 (4)	H15A—C15—H15B	107.9
C13—C4—C10	109.7 (4)	C12—C16—C15^v^	112.0 (5)
C1—C4—H4	108.5	C12—C16—H16A	109.2
C13—C4—H4	108.5	C15^v^—C16—H16A	109.2
C10—C4—H4	108.5	C12—C16—H16B	109.2
O6—C5—O8	125.7 (4)	C15^v^—C16—H16B	109.2
O6—C5—C8	118.6 (4)	H16A—C16—H16B	107.9

Symmetry codes: (i) −*x*+1, −*y*+1, −*z*+1; (ii) −*x*, −*y*+1, −*z*+1; (iii) −*x*, −*y*, −*z*; (iv) −*x*+1, −*y*+1, −*z*; (v) −*x*, −*y*+2, −*z*+1.
